# New semiquantitative parameters in digital [^18^F]FDG PET/CT improve diagnostic accuracy in suspected infective endocarditis

**DOI:** 10.1186/s41824-025-00256-6

**Published:** 2025-06-23

**Authors:** Carola Maria Bregenzer, Luisa Maria Knappe, Alexander Weissensee, Nasir Gözlügöl, Ali Afshar-Oromieh, Clemens Mingels, Christoph Gräni, Axel Rominger, Federico Caobelli

**Affiliations:** 1 Department of Nuclear Medicine, Bern University Hospital, Inselspital, University of Bern, Freiburgstrasse 18, Bern, 3000 Switzerland; 2 Department of Cardiology, University Hospital Bern, Inselspital, University of Bern, Bern, Switzerland

**Keywords:** Infective endocarditis, PET/CT, Infection, Semiquantitative parameters

## Abstract

**Background:**

The purpose of this study was to identify semiquantitative parameters of [^18^F]FDG PET/CT using a digital PET scanner, which may increase diagnostic accuracy and readers’ confidence in the diagnosis of infective endocarditis (IE).

**Results:**

Images of 82 patients undergoing [^18^F]FDG PET/CT for suspected IE were visually and semiquantitatively analyzed. Standardized uptake values (SUV) of suspected foci, also normalized to liver, mediastinum and surrounding activity were calculated. For each, best thresholds were identified to diagnose endocarditis. Final diagnosis was reached by consensus in a multidisciplinary board. Semiquantitative analysis (SUV_max_/SUV_max_ mediastinum, SUV_max_/SUV_max_ liver, SUV_peak_/SUV_peak_ mediastinum, SUV_peak_/SUV_peak_ liver, SUV_max_/SUV_mean_ liver, SUV_max_/SUV_mean_ mediastinum, SUV_max_ focus/SUV_mean_ focus, SUV_peak_/SUV_peak_ surrounding) added to visual interpretation increased sensitivity (57–86%), specificity (83–93%), PPV (64–86%), NPV (79–93%) and diagnostic accuracy (74–90%) when using best SUVs thresholds (all *p* < 0.05).

**Conclusions:**

Combining visual and semiquantitative analysis allows for a more accurate diagnosis of IE, and might be implemented into clinical routine.

## Background

In the diagnosis of infective endocarditis (IE), [^18^F]-Fluorodeoxyglucose Positron Emission Tomography ([^18^F]FDG PET/CT) has secured an important role in the latest years. In the current guidelines of the European Society of Cardiology (ESC), [^18^F]FDG PET/CT is recommended in patients with suspected prosthetic valve IE and also in patients with suspected IE on native valve, in view of its ability to detect infectious foci in the cardiac valve plane and peripheral lesions (Delgado et al. 2023).

Currently, [^18^F]FDG PET findings suggestive for IE on a visual analysis are considered a major diagnostic criterion according to the modified Duke criteria (Delgado et al. 2023). However, visual interpretation is robust if readers are expert but semiquantitative parameters may also be suggested, which are expected to increase readers’ confidence in the diagnostic workup of IE. In case of a visually detected inhomogeneous [^18^F]FDG uptake within the heart structures, different complementary semiquantitative approaches have been recently investigated (Delgado et al. 2023; Pizzi et al. 2016). While some reports focused on maximum standard uptake value (SUV_max_) of focal lesions (Salomaki et al. 2017; Jimenez-Ballve et al. 2016), other studies validated to-liver, to-blood pool and to-spleen normalized uptake values (Gazzilli et al. 2022; Pizzi et al. 2015; Swart et al. 2018).

Up to date such attempts have only been made in studies featuring analogue PET scanners. It is well known that semiquantitative parameters can vary considerably across different scanners, and this can have a major impact when new digital PET scanners are used (Fuentes-Ocampo et al. 2019). It remains unclear, whether more accurate semiquantitative parameters other than those already validated can be identified, with the goal of reducing the number of unclear [^18^F]FDG PET/CT examinations.

We hypothesized that semiquantitative parameters may help in the interpretation of [^18^F]FDG PET/CT imaging using digital PET/CT scanners. To this end, various semiquantitative parameters including standardized uptake values (SUV) of activity foci and their normalized values were determined from PET/CT examinations of patients with suspected IE. Their added value with regard to diagnostic accuracy over visual interpretation only was also tested.

## Materials and methods

### Patient population

We retrospectively investigated 82 consecutive patients with clinically suspected IE undergoing [^18^F]FDG PET/CT on a digital PET/CT scanner (featuring silicon-photomultiplier [SiPM] photodetectors). Patients were referred as part of clinical care. Patients’ characteristics are outlined in Table 1. All patients provided written informed consent for inclusion. The study was performed in accordance with the Declaration of Helsinki and the cantonal ethics committee approved the retrospective use of patients’ data (KEK-Nr. 2022–00486).

**Table 1:** Clinical data and demographic of the whole patients’ cohort

Patients *n*	82
Male sex, n(%)	64 (78)
Age (y) mean ± SD	58.5 ± 18.8
Blood culture positive, n(%)	44 (54)
Echocardiography positive, n(%)	11 (13)
Cardiac CT positive, n(%)	6 (7)
Native valve, n(%)	21 (26)
Prosthetic valve, n(%)	56 (68)
Graft composite, n(%)	36 (44)
Pacemaker, n(%)	14(17)
With Antibiotic therapy, n(%) (n)	48 (59)
Successful suppression of cardiac metabolism, n(%)	76 (93)

### Imaging protocol

Prior to the PET/CT examination, patients followed a low-carbohydrate and high-fat diet for 72 h and fasted at least 6 h. A weight-adapted dose of unfractionated Heparin (50 IU/kg) was administered 30 min prior to the radiopharmaceutical administration. Blood glucose levels were checked and were always < 10 mmol/L (180 mg/dL) prior to injection of [^18^F]FDG.

Sixty minutes after the intravenous administration of a weight-adjusted dose of 3 MBq/kg [^18^F]FDG (Range 147–300 MBq), images were acquired from skull base to mid-thigh on a digital PET/CT scanner (Biograph Vision 600 Edge, Siemens Healthineers, Erlangen, Germany) with 1.1 mm/s continuous bed motion Additionally, an electrocardiogram (ECG) gated and ungated acquisition (1-bed position) of the thorax was performed.

Images were reconstructed in 3D with a 440 × 440 × 644 matrix resulting in a voxel size of 1.65 × 1.65 × 1.65 mm^3^ with time-of-flight (TOF) and point-spread-function (PSF) after 4 iterations and 5 subsets, with a Gaussian filter (2 mm FWHM). Low-dose CT without contrast enhancement was used for attenuation correction (AC).

### Image evaluation

Visual and semiquantitative analysis of the reconstructed images were performed on a dedicated workstation (Syngo.via, Siemens Healthineers, Erlangen, Germany). An experienced technologist assessed the consistency of PET and CT data as well as their alignment, and manually corrected the co-registration, if necessary. Two experienced nuclear medicine physicians, blind to clinical data, visually evaluated the images. Discrepancies were resolved in a consensus meeting.

Based on visual appearance, images were visually rated as suggestive for IE, unclear or non-suggestive for IE. If a focus of increased uptake was detected, a semiquantitative analysis was performed. Standardized uptake values (SUV_max_, SUV_mean_, and SUV_peak_) of focal lesions were calculated by manually placing a 40%-isocontour volume-of-interest (VOI). Liver activity was calculated also by placing a 10 cm^3^ VOI in the right lobe. Blood-pool activity was calculated by placing a standard 2 × 2 pixels wide VOI centred at the lumen of the descending aorta. The activity surrounding the focal [^18^F]FDG-avid lesions was assessed by manually placing a VOI on the surrounding background without inclusion of the focal activity. Normalized parameters were calculated as ratio of SUV_max/mean/peak_ of focal lesions to liver, to blood pool and to surrounding background. A ratio between SUV_max_ and SUV_mean_ of the focal lesion was calculated, as surrogate marker for heterogeneity.

### Final diagnosis

The final clinical diagnosis was reached in a multidisciplinary board, consisting of cardiologists, expert physicians in infectious diseases, radiologists and nuclear medicine physicians.

### PET-derived diagnosis

PET-derived diagnosis was first reached on visual interpretation only. In this case, unclear PET results were always rated as false (i.e. false negative if the final diagnosis of IE was confirmed and false positive if it was rejected). Then, the diagnostic performance of various semiquantitative PET/CT parameters (SUV_max/mean/peak_, each also normalized to SUV_max/mean/peak_ of liver/mediastinum/surrounding activity, as described above) was assessed, with the determination of the best thresholds for each parameter. Finally, the diagnostic accuracy of combined visual and semiquantitative analysis was assessed. To that end, PET was rated positive if the visual interpretation was definitely pathological and/or if at least one of the semiquantitative parameters was above the defined thresholds.

### Statistical analysis

Statistical analysis was performed using IBM SPSS (Version 28.0.1.1, IBM Corp. Armonk, NY, USA) and R (www.r-project.org). Continuous variables were compared in patients with and without IE using Mann-Whitney U-Test. Categorical variables were compared by Fischer’s Exact test. Diagnostic accuracy of all semiquantitative parameters was tested by receiver-operator curves (ROC) analysis, using Youden’s Index to identify the best threshold values Finally, Chi Square analysis and Compbdt in R was used to calculate the diagnostic performance. *P* values < 0.05 were considered statistically significant.

## Results

The study cohort included 82 consecutive patients (64 males, 78%, age 58.5 ± 18.8 years) After discussion in the multidisciplinary board, a final diagnosis of IE was made in 31 (37.8%) patients, while 51 were classified as IE negative. Positive blood cultures during the period before examination were reported in 44 patients. Patients received antibiotic therapy for 13.5 days median before undergoing PET/CT examination (interquartile range 14.25 days).

### Visual interpretation

**Fig. 1:**
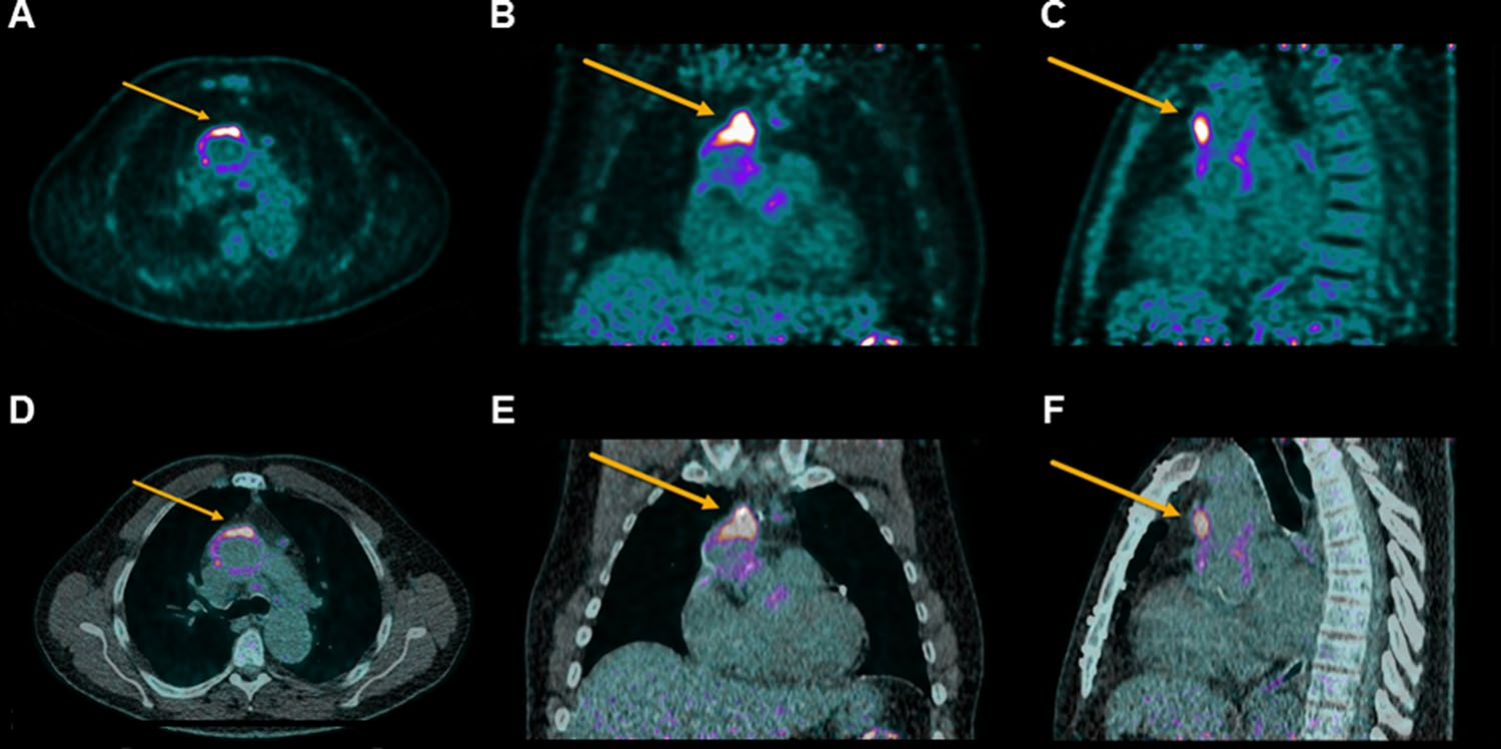
Abnormal, inhomogenous, focal uptake of the ascending aorta on PET only (A-C) and on PET/CT (D-F) in axial (A&D), coronal (B&E) and sagittal (C&F). Lesion uptake parameters were SUV_max_ 18.5, SUV_mean_ 3.0 SUV_peak_ 11.1

Abnormal visual uptake was observed in 32 patients (39%)with a total of 49 hypermetabolic foci. In 15/32 patients only one focus was present while two foci were seen in 15/32 patients (Fig. 1), and 3 foci in 2/32 patients. The visual analysis led to PET-derived diagnosis of probable IE in 19 (23.2%) patients, rejected IE in 47 (57.3%) and unclear in 16 (19.5%).

### Semiquantitative parameters

For all 16 patients with unclear diagnosis after visual analysis adding semiquantitative parameters to the interpretation led to reach a definite PET-derived diagnosis in all of them (12 more confirmed and 4 more rejected IE). SUV_max_ liver was higher in patients with IE (4.0 ± 0.1 vs. 3.9 ± 1.4, *p* = 0.005). Same held true for surrounding SUV_max_ (5.1 ± 2.7 vs. 4.9 ± 2.3, *p* = 0.008).

Normalized values were different in patients with and without IE: to-mediastinum SUV_max_ (3.8 ± 0.4 vs. 2.3 ± 0.6, *p* = 0.002), to-liver SUV_max_ (2.4 ± 0.2 vs. 1.6 ± 0.5, *p* = 0.002), to-mediastinum SUV_peak_ (2.2 ± 0.2 vs. 1.7 ± 0.5, *p* = 0.002), to-liver SUV_peak_ (1.9 ± 0.2 vs. 1.4 ± 0.4, *p* = 0.004), to-liver SUV_max_/SUV_mean_ (2.0 ± 0.6 vs. 1.2 ± 0.4, *p* = 0.004), to-mediastinum SUV_max_/SUV_mean_ (2.5 ± 0.3 vs. 1.4 ± 0.5, *p* = 0.005), SUV_max_/SUV_mean_ focus (3.3 ± 0.7 vs. 2.0 ± 0.7, *p* = 0.005), SUV_peak_/SUV_peak_ surrounding (1.8 ± 0.5 vs. 1.2 ± 0.2, *p* = 0.01). Differences in all semiquantitative parameters and their diagnostic performance evaluated by means of ROC curves are depicted in Table 2.

**Table 2:** Age and semiquantitative parameters in patients with confirmed and rejected infective endocarditis. AUC is also displayed

	with IE	without IE	*p*	AUC CI (95%)
**Age**	56.7 ± 13.9	56.0 ± 20.6	0.330	**-**
**SUVmax/ SUVmax mediastinum**	3.8 ± 0.4	2.3 ± 0.6	0.002	**0.88** ( 0.74 - 1.0)
**SUVmax/ SUVmax liver**	2.4 ± 0.2	1.6 ± 0.5	0.002	**0.87** (0.71 - 1.0)
**SUVpeak/ SUVpeak mediastinum**	2.2 ± 0.2	1.7 ± 0.5	0.002	**0.87** (0.72 - 1.0)
**SUVpeak/ SUVpeak liver**	1.9 ± 0.2	1.4 ± 0.4	0.004	**0.85** (0.7 - 1.0)
**SUVmax/ SUVmean liver**	2.0 ± 0.6	1.2 ± 0.4	0.004	**0.84** (0.68– 1.0)
**SUVmax/ SUVmean mediastinum**	2.5 ± 0.3	1.4 ± 0.5	0.005	**0.84** (0.67– 1.0)
**Focus SUVmax/ SUVmean focus**	3.3 ± 0.7	2.0 ± 0.7	0.005	**0.83** (0.66– 1.0)
**SUVpeak/ SUVpeak surrounding**	1.8 ± 0.5	1.2 ± 0.2	0.01	**0.81** (0.66– 0.96)
**SUVmax/SUVmean surrounding**	2.1 ± 0.9	1.6 ± 0.4	0.001	**0.68** (0.46– 0.9)
**SUVmax liver**	4.0 ± 0.1	3.9 ± 1.4	0.005	**-**
**SUVmax surrounding**	5.1 ± 2.7	4.9 ± 2.3	0.008	**-**
**SUVmax focus/SUVmax surrounding**	2.2 ± 1.0	1.3 ± 0.3	0.17	**0.79** (0.63– 0.94)
**SUVmax focus**	9.7 ± 0.8	6.3 ± 3.0	0.019	**0.78** (0.56– 1.0)
**SUVpeak surrounding**	3.1 ± 0.7	3.4 ± 1.2	0.023	**-**
**SUVmax mediastinum**	2.6 ± 0.1	2.7 ± 0.7	0.042	**-**
**SUVpeak focus**	5.3 ± 0.4	3.9 ± 1.7	0.050	**0.73** (0.51– 0.96)
**(SUVmax focus/SUVmax surrounding)/(SUVmean focus/ SUVmean surrounding)**	1.6 ± 0.4	1.2 ± 0.3	0.090	**0.70** (0.52– 0.89)
**SUVpeak mediastinum**	2.4 ± 0.1	2.3 ± 0.5	0.120	**-**
**SUVmean surrounding**	2.3 ± 0.3	2.9 ± 1.0	0.193	**-**
**SUVpeak liver**	2.9 ± 0.1	2.8 ± 0.6	0.229	**-**
**SUVmean focus/SUVmean surrounding**	1.3 ± 0.3	1.2 ± 0.3	0.338	**0.62** (0.38– 0.85)
**SUVmean mediastinum**	1.9 ± 0.3	2.0 ± 0.5	0.639	**-**
**SUVmean focus**	3.0 ± 0.4	3.3 ± 1.2	0.728	**0.54** (0.32– 0.77)
**SUVmean/SUVmean mediastinum**	1.5 ± 0.0	1.6 ± 0.3	0.794	**0.53** (0.32– 0.74)
**SUVmean liver**	2.4 ± 0.2	2.3 ± 0.4	0.895	**-**
**SUVmean/SUVmean liver**	1.3 ± 0.3	1.4 ± 0.3	0.965	**0.50** (0.29– 0.70)

### Best thresholds

**Fig. 2:**
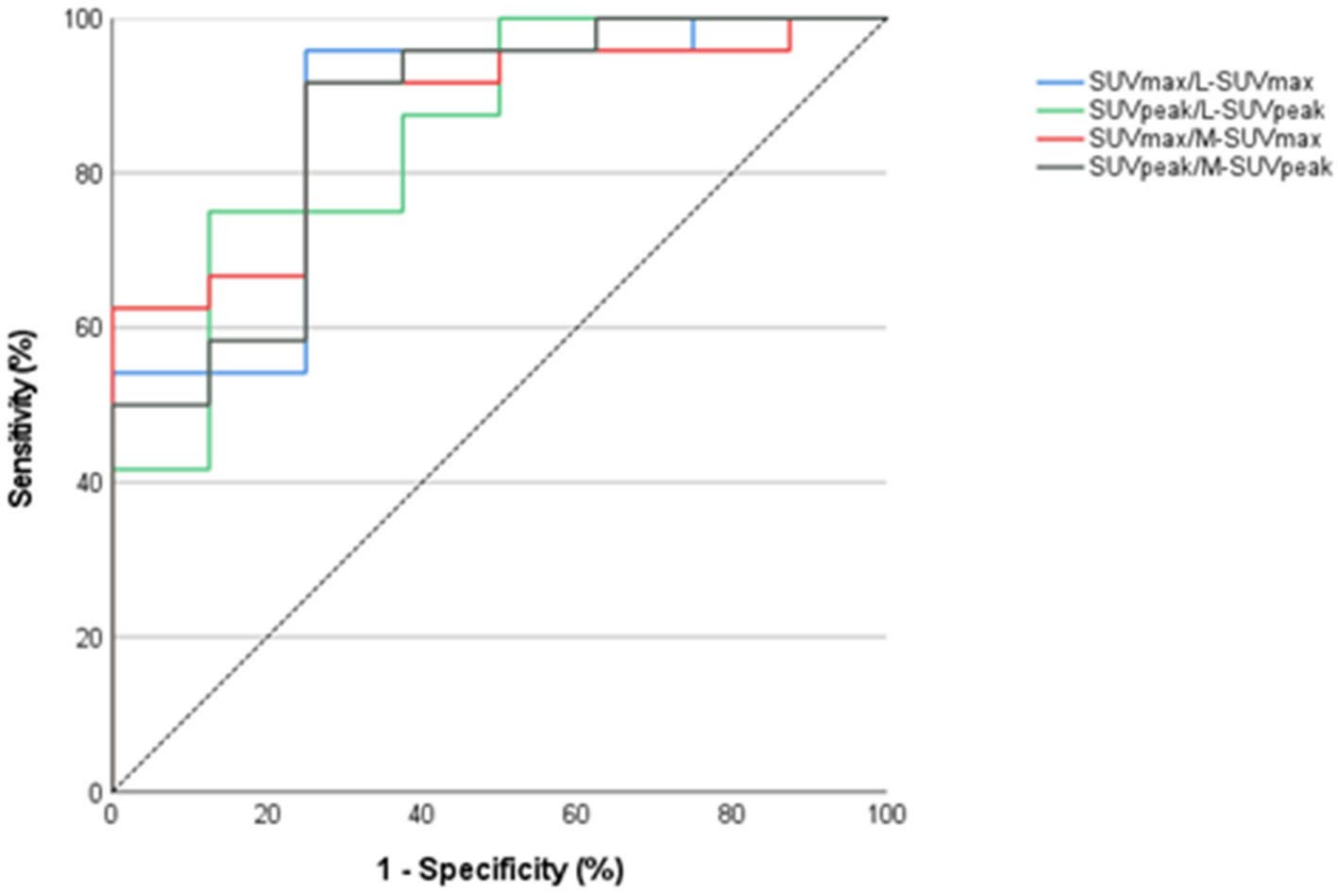
Best normalized parameters with (AUC): SUV_max_/SUV_max_ mediastinum (M-SUV_max_, 0.88), SUV_max_/SUV_max_ liver (L-SUV_max_. 0.87), SUV_peak_/SUV_peak_ mediastinum (M-SUV_peak_, 0.87), SUV_peak_/SUV_peak_ liver (L-SUV_peak_ 0.85)

**Fig. 3:**
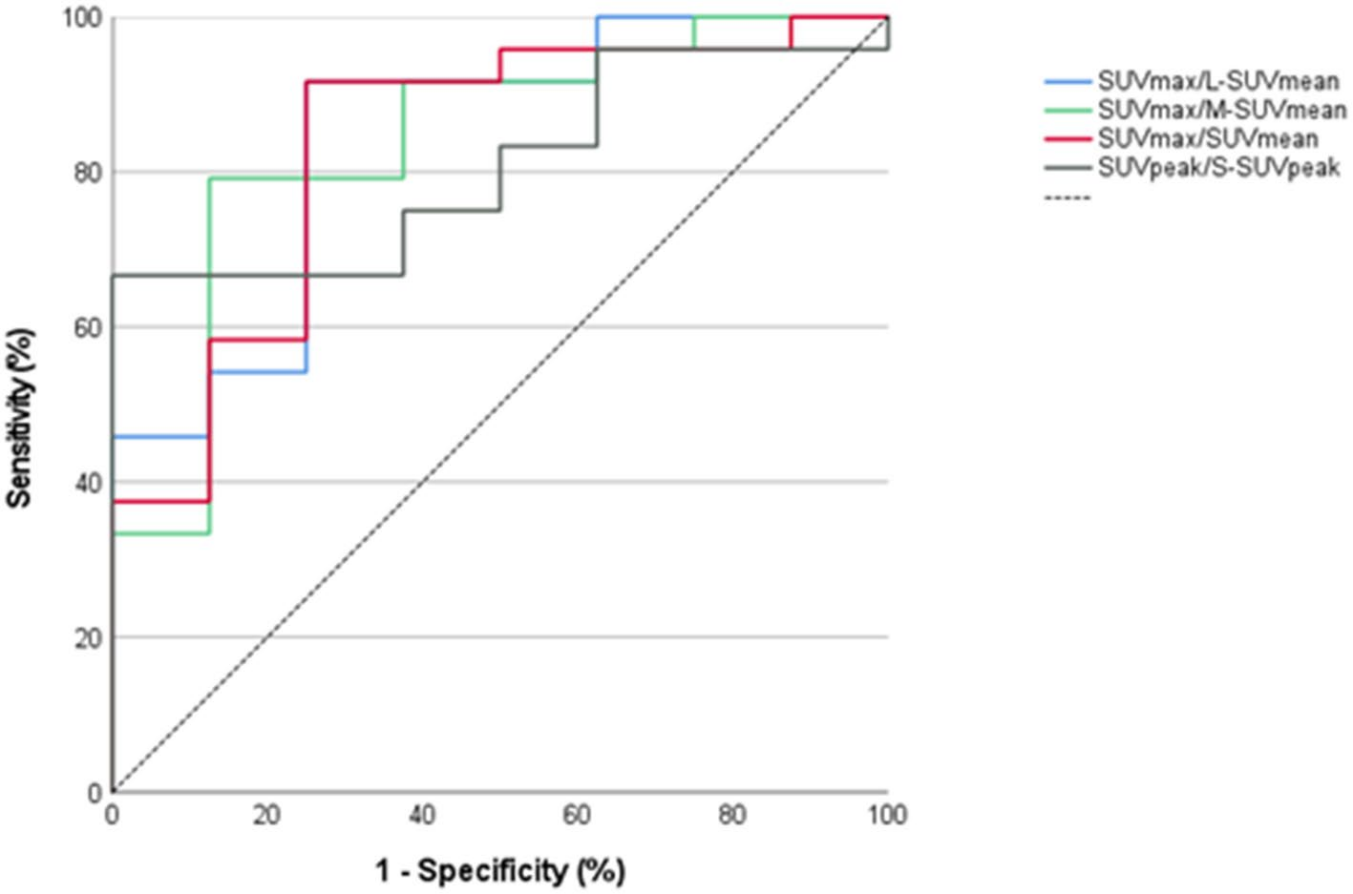
ROC curves of the parameters with the highest AUC > 0.8: SUV_max_/SUV_mean_ liver (L-SUV_mean_, 0.84)), SUV_max_/SUV_mean_ mediastinum (M-SUV_mean_, 0.84), SUV_max_/SUV_mean_ (focus, 0.83), SUV_peak_/SUV_peak_ surrounding (S-SUV_peak_, 0.81)

For all semiquantitative parameters, a ROC analysis was performed. Among the semiquantitative parameters with the highest accuracy (AUC ≥ 0.8), best thresholds for the diagnosis of IE were calculated by means of Youden’s index. This applied to 8 parameters (Table 2; Figs. 2 and 3). Best thresholds are displayed in Table 3.

**Table 3:** Semiquantitative parameters with highest AUC, with best Threshold and sensivitity/specificity values

	Threshold	Sensitivity	Specificity
**SUVmax/SUVmax mediastinum**	2.54	91.7%	75.0%
**SUVmax/SUVmax liver**	1.64	95.8%	75%
**SUVpeak/SUVpeak liver**	1.69	75.0%	87.5%
**SUVpeak/SUVpeak mediastinum**	1.67	91.7%	75.0%
**SUVmax/SUVmean liver**	1.24	91.7%	75.0%
**SUVmax/SUVmean mediastinum**	1.58	79.2%	87.5%
**SUVmax/SUVmean**	1.86	91.7%	75.0%
**SUVpeak/SUVpeak surrounding**	1.41	66.7%	100%

### Comparison between visual and semiquantitatively assisted analysis

**Fig. 4:**
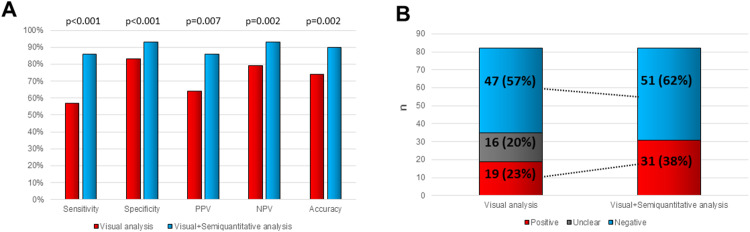
A: Sensitivity, specificity, PPV, NPV and accuracy with visual analysis only (red). Together with semiquantitative analysis (blue) all five parameters could be improved. B: Numbers with (percentage) Left column: Positive (red), unclear (grey) and negative (blue) diagnosis of infective endocarditis on PET/CT with visual analysis. Adding semiquantitative analysis (right column) increased the number of positive (red) and negative (blue) diagnosis

With visual analysis only, sensitivity was 57% (confidence interval (CI) 95%: 39–74), specificity 83% (CI 95%: 71–91), positive predictive value (PPV) 64% (CI 95%: 45–80), negative predictive value (NPV) 79% (CI 95%: 67–88) and accuracy 74% for the final consensus diagnosis of IE. Adding semiquantitative analysis allowed to increase diagnostic performance. Sensitivity increased to 86% (CI 95%: 69–95), specificity to 93% (CI 95%: 83–97), PPV to 86% (CI 95%: 69–95), NPV to 93% (CI 95%: 83–97), and accuracy to 90% (Fig. 4A-B). Sensitivity and specificity were statistically significant increased with *p* < 0.001 (sensitivity CI 95%: 8–45; specificity CI 95%: (-1)-19) as well as positive (*p* = 0.007 (CI 95%:6–37)) and negative predictive value (*p* = 0.002 (CI 95%: 5–22)).

## Discussion

We demonstrated that semiquantitative parameters assessed by digital [18 F]FDG PET/CT improves reader’s confidence and diagnostic accuracy compared to visual analysis only. Specifically, normalized semiquantitative values yield highest accuracy.

Other papers investigated a possible role for semiquantitative analysis to assist in the PET-derived diagnosis of IE. In a previous study, a cut-off of SUV_max_ 4.0 resp. 5.5 for the [^18^F]FDG focus was suggested (Salomaki et al. 2017; Jimenez-Ballve et al. 2016). Of note, specificity in this paper was inferior to that obtained with two of our thresholds with 0.8 ≥ AUC (e.g. SUV_peak_/SUV_peak_ liver, SUV_max_/SUV_mean_ mediastinum where a specificity of 87.5% could be reached compared to a specificity of 80%). Specificity is often suboptimal with [^18^F]FDG PET/CT, as [^18^F]FDG uptake can be present in patients with chronic inflammation without infection and especially in patients with previous cardiac surgery (Rouzet et al. 2014). The results of our study may therefore indicate that the two abovementioned parameters yield high -specificity, and this strengthen the concept that the activity within the suspected infectious focus should be always evaluated compared to background activities (i.e. in the liver and in the bloodpool).

Gazzilli et al.(Gazzilli et al. 2022) suggested a Deauville score like scale evaluating [^18^F]FDG activity in the focus in relation to that of bone marrow, spleen and liver. While this approach provided interesting insights and yielded good diagnostic accuracy, still a possible limitation was the possible interference from other inflammatory conditions. In this regard, the results of our study show that other semiquantitative parameters may be more accurate in the definition of IE, and that this advantage is bolstered if visual and semiquantitative evaluation are performed together.

Not only individual patient’s characteristics and clinical conditions (e.g. previous cardiac surgery, general inflammatory status), but also semiquantitative parameters seem to be different depending on the patient cohort. In a study featuring analogue PET/CT scanners, Pizzi et al. (Pizzi et al. 2015) proposed SUV_max_ focus/SUV_mean_ bloodpool with a cut-off of 1.68 (sensitivity 90.74%, specificity 76.32%), while Swart et al. (Swart et al. 2018) proposed for the same parameter a cut-off of 2.1 (sensitivity 75%, specificity 86%, AUC 0.81). We also investigated the best threshold for this parameter in our cohort, and found that, using a digital PET/CT, the best threshold is 1.58 (sensitivity 79.2%, specificity 87.5%). Of note, sensitivity and specificity are similar compared to Swart et al. (Swart et al. 2018) using a different cut-off. Hence, it can be maintained, that as it happens in other areas of PET imaging (Knappe et al. 2023), semiquantitative parameters should be validated separately for analogue, digital and long-axial field-of-view (LAFOV) PET scanners. The novelty of our study is investigating in semiquantitative parameters on a digital PET scanner in patients with infective endocarditis. In this regard, our study provides for the first time, the impact of semiquantitative parameters on a digital PET scanner in IE workup. Of note, digital scanners will conceivably represent the state-of-the-art of PET imaging, and the definition of thresholds able to accurately diagnose IE on these scanners is highly anticipated.

The results of our study may suggest to reconsider the criteria used for PET evaluation. To now, visual analysis for intense focal or heterogenous prosthetic/periprosthetic uptake is recommended (Delgado et al. 2023). However, it may be suggested that in uncertain cases a semiquantitative analysis be implemented, in order to increase readers’ confidence and diagnostic accuracy. Of note, a semiquantitative analysis may not be necessary in case of clearly unremarkable or pathological visual findings on PET. In our cohort, PET was firstly classified as unclear after visual analysis in 16 patients, but adding semiquantitative parameters to the interpretation led to reach a definite PET-derived diagnosis in all of them (12 more confirmed and 4 more rejected IE). The fact that diagnostic accuracy increased with this approach gives more reliance in adopting our proposed parameters in clinical practice.

In this regard, a combination of two or more parameters may be considered. For example, the parameter SUV_peak_/SUV_peak_ surrounding (threshold 1.41, sensitivity 66.7%) showed a specificity of 100%. Hence, its combination with a highly sensitive parameter such as SUV_max_/SUV_max_ liver (95.8% sensitivity) may yield highest accuracy in clinical practice.

### Limitations

The main limitation of our study is its retrospective and single-center nature. As such, our patients’ sample is small, heterogeneous with regard to therapy and surgical procedures. Furthermore, the final clinical diagnosis was based on a multidisciplinary consensus meeting rather than on pathologic confirmation of bacterial specimens. However, multidisciplinary board is now considered as the most accurate gold standard in daily clinical practice for the assessment of IE (Delgado et al. 2023). Second, a subgroup analysis for patients with native and prosthetic valves was not feasible. It should be noted, that the diagnosis of IE in native valves is less challenging and typically relies on echocardiographic findings (Caobelli et al. 2017). In our cohort, the initial clinical assessment was equivocal for all patients. In some patients previous Cardiac CT angiography was performed with primarily negative or unclear results. Hence, the gain in diagnostic accuracy observed in our study when adding semiquantitative parameters is conceivably not affected by this drawback. Finally, some patients were on antibiotic treatment at the time of PET/CT. Often, beginning of antibiotic therapy cannot be postponed due to severe clinical conditions. Antibiotic therapy could influence diagnostic accuracy especially with false negative scans, although in previous studies capitalizing on white blood cell scintigraphy, such negative effect seems to be low (Caobelli et al. 2017). Furthermore, it seems that also the duration of antibiotic treatment has little– if any– effect in [18 F]FDG PET imaging (Swart et al. 2018; Primus et al. 2022).

While our study suggests the applicability of semiquantitative parameters in the diagnostic workup of IE, larger, prospective studies on digital PET scanners are needed to validate our proposed thresholds and to assess their impact on therapy decision.

## Conclusion

Incorporating semiquantitative parameters into visual assessments enhances the diagnostic accuracy of digital [^18^F]FDG PET scans for patients with suspected infective endocarditis (IE). Therefore, routinely combining visual and semiquantitative interpretations can lead to a more precise evaluation, ultimately benefiting patient outcomes.

## Data Availability

Data will be made available upon reasonable request.

## Abbrevations

AC: attenuation correction;
ECG: electrocardiogram;
ESC: European Society of Cardiology;
IE: infective endocarditis;
LAFOV: long-axial field-of-view;
PSF: point-spread-function;
ROC: receiver-operator curves;
SUV: standardized uptake values;
TOF: time-of-flight;
